# Cell-free DNA promotes malignant transformation in non-tumor cells

**DOI:** 10.1038/s41598-020-78766-5

**Published:** 2020-12-10

**Authors:** Aline Gomes de Souza, Victor Alexandre F. Bastos, Patricia Tieme Fujimura, Izabella Cristina C. Ferreira, Letícia Ferro Leal, Luciane Sussuchi da Silva, Ana Carolina Laus, Rui Manuel Reis, Mario Machado Martins, Paula Souza Santos, Natássia C. Resende Corrêa, Karina Marangoni, Carolina Hassibe Thomé, Leandro Machado Colli, Luiz Ricardo Goulart, Vivian Alonso Goulart

**Affiliations:** 1 Laboratory of Nanobiotechnology, Institute of Biotechnology, Federal University of Uberlândia, Uberlândia, MG Brazil; 2 Department of Medical Imaging, Hematology, and Oncology, Ribeirão Preto Medical School - University of São Paulo, Ribeirão Preto, Brazil; 3 Molecular Oncology Research Center, Barretos Cancer Hospital, Barretos, SP Brazil; 4 Department of Medical Microbiology and Immunology, University of California-Davis, Davis, USA; 5 Life and Health Sciences Research Institute (ICVS), Medical School, University of Minho, Braga, Portugal; 6 3ICVS/3B’s-PT Government Associate Laboratory, Braga, Portugal; 7 Center for Cell Based Therapy, Hemotherapy Center of Ribeirão Preto, Ribeirão Preto, SP Brazil

**Keywords:** Cancer, Cell biology, Molecular biology, Oncology, Urology

## Abstract

Cell-free DNA is present in different biological fluids and when released by tumor cells may contribute to pro-tumor events such as malignant transformation of cells adjacent to the tumor and metastasis. Thus, this study analyzed the effect of tumor cell-free DNA, isolated from the blood of prostate cancer patients, on non-tumor prostate cell lines (RWPE-1 and PNT-2). To achieve this, we performed cell-free DNA quantification and characterization assays, evaluation of gene and miRNA expression profiling focused on cancer progression and EMT, and metabolomics by mass spectrometry and cellular migration. The results showed that tumor-free cell DNA was able to alter the gene expression of *MMP9* and *CD44*, alter the expression profile of nine miRNAs, and increased the tryptophan consumption and cell migration rates in non-tumor cells. Therefore, tumor cell-free DNA was capable of altering the receptor cell phenotype, triggering events related to malignant transformation in these cells, and can thus be considered a potential target for cancer diagnosis and therapy.

## Introduction

Before the DNA structure was described by Watson and Crick in 1953, Mandel and Métais in 1948 reported the presence of DNA in the plasma of individuals^1^. This free circulating DNA has been described as circulating cell-free DNA (cfDNA). cfDNA refers to extracellular DNA fragments that can be detected in different biological fluids^2^. Currently, the main application of cfDNA is in the diagnosis of different neoplasms, since changes present in this molecule may indicate the presence of cancer cells^3^. The source of cfDNA in circulation is still poorly understood. Studies indicate that cellular events such as apoptosis, necrosis and cell secretion can affect the amount of circulating cfDNA fragments^4,5^. In cancer patients, epigenetic alterations, mutations, and over-expression of certain genes in cfDNA are some of the main investigations being done to detect biomarkers in this type of liquid biopsy^6,7^.

In addition to its application as a potential biomarker, studies involving healthy and neoplastic participants are being conducted in an attempt to understand the role of cfDNA in biological events, as demonstrated by Zinkova et al.^8^. The authors, found that cfDNA from healthy participants had a role in regulating the immune response. García-Olmo et al.^10^, in clinical studies from colon cancer patients, also found evidence of gene transfer and cell transformation triggered by nucleic acids isolated from plasma^9,10^. Therefore, they proposed that plasma cfDNA participates in tumorigenesis and in the development of receptor cell transformation metastases, which they called “genometastasis”^9^.

These studies and advances in molecular biology have allowed us to verify the functionality of cfDNA in cellular communication and, consequently, in the development and progression of diseases such as cancer^11,12^.

Epithelial–mesenchymal transition (EMT) is one of the most discussed biological events in tumorigenesis and metastases, however, the molecular mechanisms governing EMT and its crosstalk with cfDNA remain poorly understood^13,14^.

The in vitro analyses evaluating the role of cfDNA in tumorigenesis is poorly explored. Therefore, this work evaluated for the first time the potential of cfDNA, isolated from prostate cancer (PCa) patients, in the malignant transformation of non-tumor cells in vitro. We have detected malignant alterations in non-tumor cells post-treatment with tumor cfDNA isolated from plasma.

## Results

### Plasma cfDNA of prostate cancer patients is more fragmented and in higher concentration

**Figure 1:**
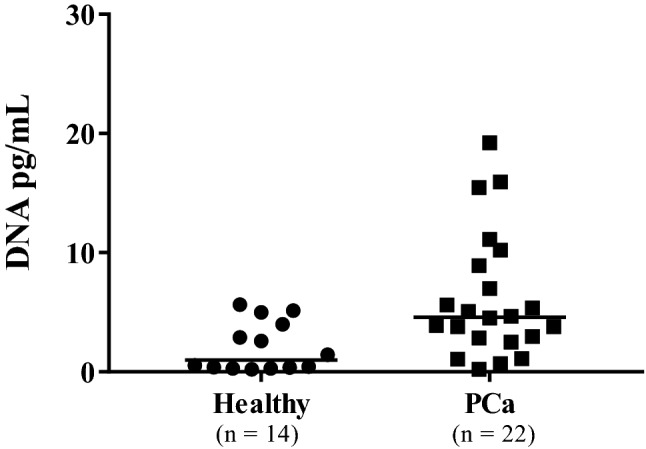
Quantification of plasma cfDNA in prostate cancer (PCa) patients measured by qPCR. Data are reported as medians (horizontal line). (*p < 0.05; Mann–Whitney *U*-test).

Fragment size and levels of cfDNA were measured in plasma samples from cancer patients (n = 22) and from healthy controls (n = 14). The cfDNA fragment sizes in healthy control samples were longer (174–7387 bp range) than the fragment sizes in T1/T2 patients (157–4912 bp range) and T3/T4 patients (range, 29–53 bp), (Supplementary Fig. S1). Moreover, the cfDNA concentration in healthy control samples (median 2.1 pg/mL plasma, n = 14) was significantly lower than in cancer prostate samples (median = 6.2 pg/mL plasma; n = 22, p < 0.05) (Fig. 1). Individual quantification of cfDNA from patients was performed in duplicates (Supplementary Fig. S2A,B). Moreover, no statistical difference was observed comparing cfDNA levels between the tumor stages and the Gleason score (Supplementary Fig. S3A,B).

### Circulating tumor cfDNA increases gene expression related to epithelial-mesenchymal transition (EMT)

**Figure 2:**
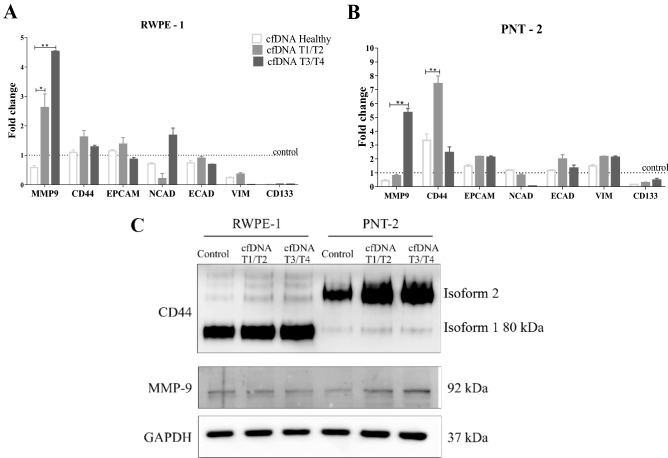
Effect of plasma cfDNA from prostate cancer patients on the expression of genes associated with the epithelial-mesenchymal transition in RWPE-1 (**A**) and PNT-2 (**B**) cells. Twenty nanograms of cfDNA were incubated with 1 × 10^5^ cells for 24 h. Gene expression was determined by RT-qPCR and data are reported as fold change increase of gene expression compared to control cells untreated with cfDNA. The dotted line represents control expression set to 1. Results are expressed as mean ± S.D, (*p < 0. 05 and **p < 0.01; Kruskal–Wallis test). (**C**) Western blotting analyses of CD44 isoforms and MMP9 in RWPE-1 and PNT-2 cells. The GAPDH protein was used as an endogenous control. The full-length blots are presented in Supplementary Fig. S4.

Since EMT is a very important process in the progression of cancer, we compared the EMT-linked gene expression in RWPE-1 and PNT-2 cell lines treated with tumor cfDNA using qPCR. The data of qPCR shows alterations in the expression of *MMP9* and *CD44* genes in PNT-2, and *MMP9* gene in RWPE-1 cells. There was a difference of gene expression between T1/T2 and T3/T4 cfDNA treatments. RWPE-1 cells presented an increased expression of *MMP9* in both treatments with tumor isolated cfDNA (Fig. 2A). In the PNT-2, *MMP9* expression was significantly increased when treated with T3/T4 cfDNA, and similar results were observed for *CD44* in the treatment with T1/T2 cfDNA (Fig. 2B). Levels of this protein (CD44 isoform 2) also increased in the treatment with tumor cfDNA in PNT-2, however, MMP9 protein levels have not been changed (Fig. 2C). The expression of other EMT genes did not show changes for both cell lines. These data strongly suggested that independent molecular signaling pathways might be regulated according to stimuli via tumor cfDNA. Moreover, the tumor stage from which the cfDNA was isolated may also be a limiting factor.

### cfDNA alters miRNA regulation in non-tumor cells

**Figure 3:**
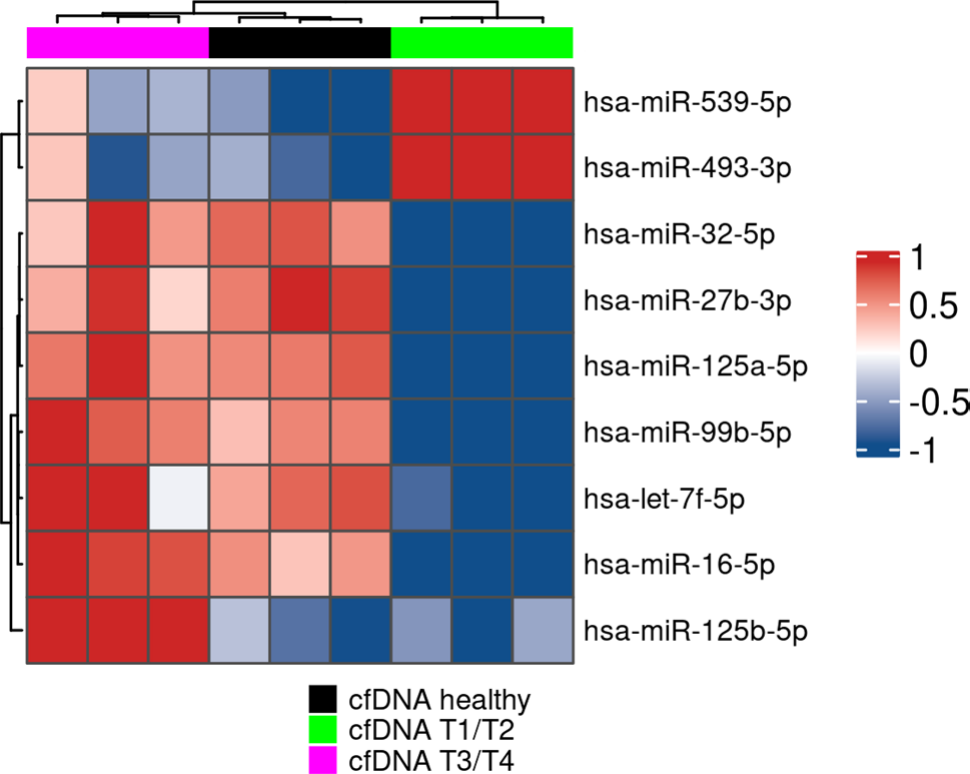
Hierarchical clustering of the differentially expressed miRNAs after treatment with cfDNA healthy, cfDNA T1/T2, and cfDNA T3/T4 using NanoString nCounter miRNA Expression Assays. Red: up-regulated, blue: down-regulated. (padj < 0.05, ANOVA; FC > 1.5).

Considering that the PNT-2 cell line presented alterations in more genes (*MMP9* and *CD44*), we further performed a miRNA expression analysis. Out of the 800 miRNAs analyzed, nine miRNAs presented significant differential expression for one of the three groups analyzed with a fold change of at least 1.5. The results showed that the treatment with T1/T2 cfDNA and T3/T4 cfDNA promoted deregulation of miRNAs. In the treatment with T1/T2 cfDNA, hsa-miR-539-5p and hsa-miR-493-3p were upregulated, while the hsa-miR-32-5p, hsa-miR-27b-3p, hsa-miR-125a-5p, hsa-miR-99b-5p, hsa-let-7f.-5p and hsa-miR-16-5p were down-regulated. However, in the treatment with T3/T4 cfDNA only hsa-miR-125b-5p was up-regulated (Fig. 3, and supplementary table S1) and no statistical significance was observed in the eight miRNAs when comparing the treatments between T3/T4 cfDNA and healthy cfDNA groups, p_adj_ > 0.05. A comparative analysis was also performed between healthy cfDNA and non-treated cells, and no statistical difference was observed between these groups (supplementary table S2).

### Transformation of a normal cell with T3/T4 patient cfDNA stimulates the uptake of tryptophan from the medium

**Figure 4:**
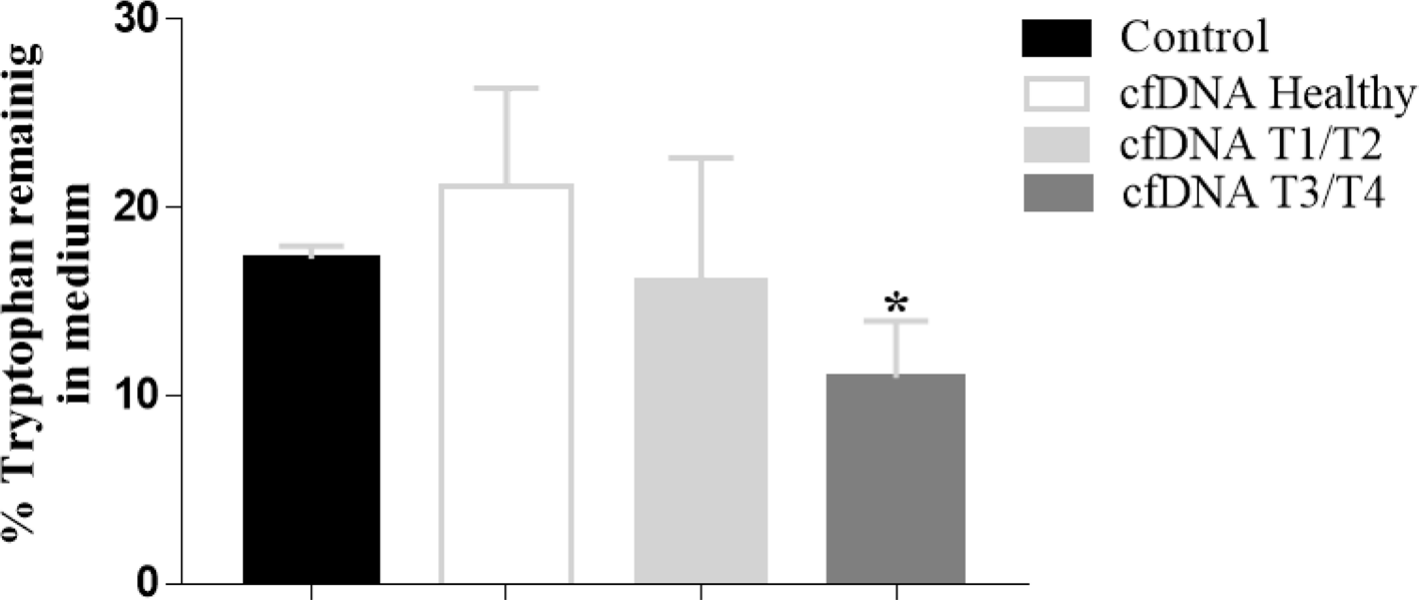
Treatment of RWPE-1 cells with T3/T4 patient cfDNA stimulated the uptake of tryptophan from the medium. Twenty nanograms of cfDNA were incubated with 1 × 10^5^ cells in medium containing 5 mM tryptophan. The tryptophan levels in medium were determined by metabolomic analysis. The medium of cells not-treated with cfDNA was used as a control. Data are reported as means ± SD of tryptophan levels (%). (*p < 0.05; Dunn's multiple comparison test).

Data obtained by metabolomic analysis showed an abundant percentage of 142 metabolites present on the PNT-2 cell line, as well as 108 metabolites in the RWPE-1 cell line (Supplementary Tables S3, S4). In this analysis the essential amino acid tryptophan was identified (Supplementary Fig. S5) as the metabolite with statistically significant alteration. This metabolite showed reduced supernatant levels in cells treated with T3/T4 cfDNA compared to the cells treated with cfDNA isolated from healthy subjects and the control, p < 0.05 (Fig. 4).

### Tumor cfDNA increased mobility of non-tumor cells

**Figure 5:**
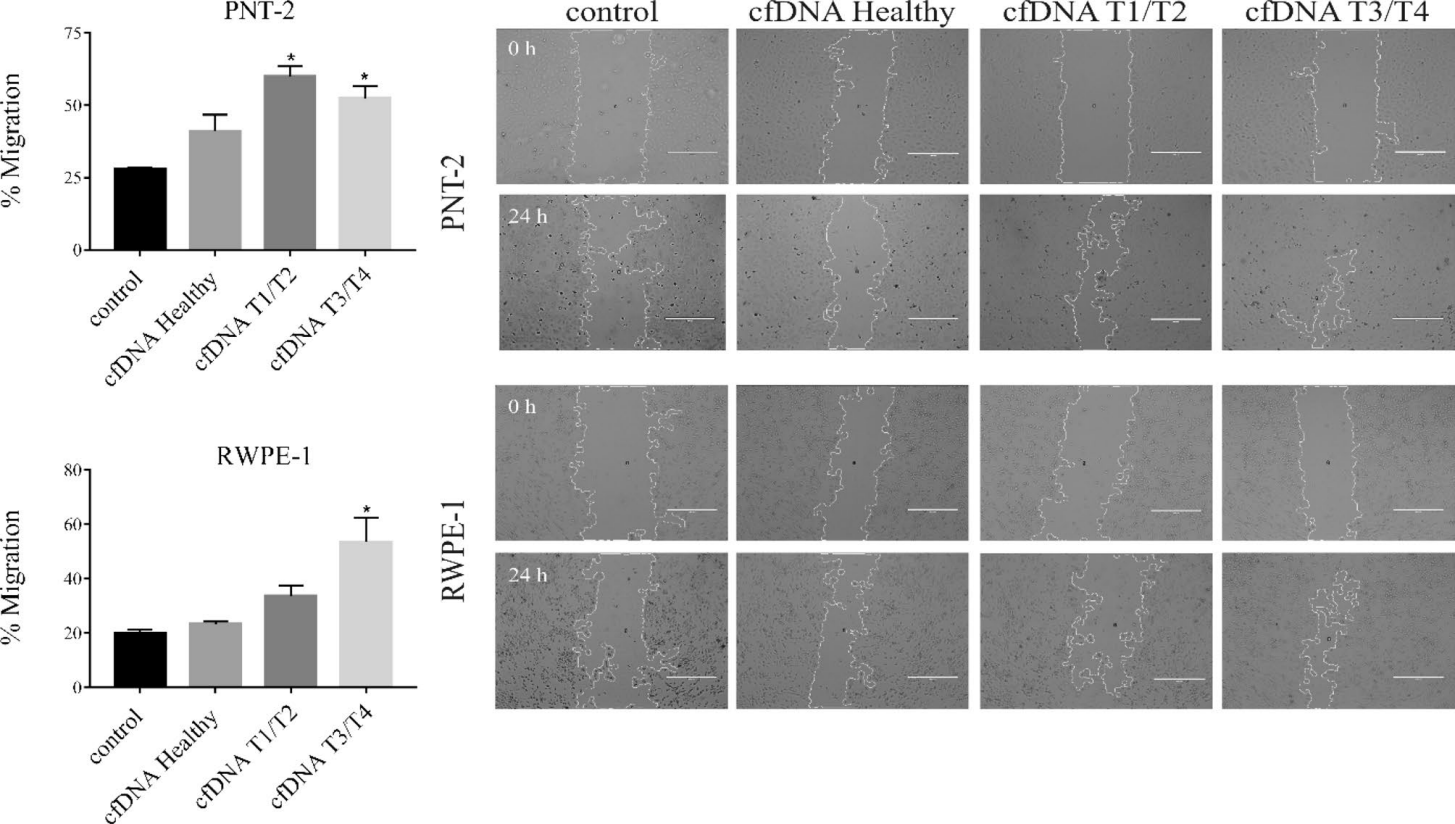
Evaluation of the influence of cfDNA treatment on migration. PNT-2 (**A**) and RWPE-1 (**B**) cell lines were treated with cfDNA healthy, cfDNA T1/T2, cfDNA T3/T4. Twenty nanograms of cfDNA was incubated with 1 × 10^4^ cells for 24 h. Percentage of migraton was determined by calculated area in ImageJ software. Data are expressed as the mean ± SEM of at least three independent experiments (*p < 0. 05; Kruskal–Wallis test).

**Figure 6:**
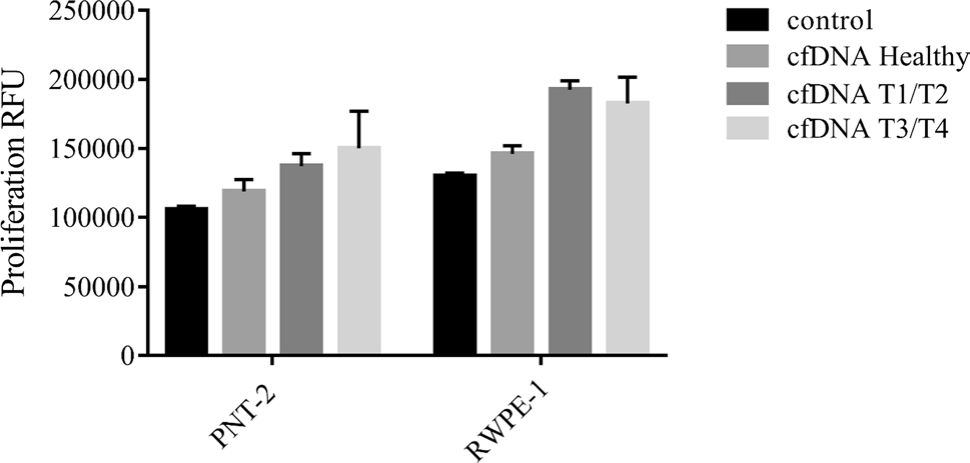
Evaluation of the influence of cfDNA treatment on proliferation. PNT-2 and RWPE-1 cell lines were treated with cfDNA healthy, cfDNA T1/T2, and cfDNA T3/T4**.** Twenty nanograms of cfDNA were incubated with 1 × 10^4^ cells for 24 h. The proliferation was measured by relative fluorescence units. Data are expressed as the mean ± SEM of at least three independent experiments. No statistical differences were observed (*p < 0. 05; Kruskal–Wallis test).

Based on previous results, we investigated the cellular migration and proliferation. We observed that cell migration was enhanced after treatment with T3/T4 cfDNA in both cell lines (RWPE-1 and PNT-2). The treatment with T1/T2 cfDNA also increased the migration of PNT-2 cells (Fig. 5). However, the cell proliferation did not show statistical differences in any of the treatments with cfDNA, p > 0.05 (Fig. 6).

## Discussion

To our knowledge, this work is the first to address the study of prostatic tumor cfDNA on prostatic non-tumor cells based on the genometastasis theory. This theory highlights the transference of oncogenes from tumor cells to susceptible healthy cells in other organs, through the circulatory system. This horizontal transference of oncogenes can trigger various intracellular mechanisms and alter the biology of the receptor cell^9,10,15^.

Circulating cfDNA has recently received special attention in the field of cancer diagnostics. Different studies have been conducted for the implementation of tumor cfDNA as a tool to detect diseases such as cancer^4,16^. Although the source of cfDNA in the cellular environment is still poorly understood, the presence of this molecule in different body fluids drove us to investigate its biological role in communication and cellular behavior. However, few studies have evaluated the function of tumor cfDNA in tumor biology.

The malignant transformation of cells is a multi-step process and requires a set of cellular and microenvironmental changes^17–20^. Thus, we investigated several different molecular and biological aspects of the cells that are evaluated in the tumorigenesis process, these include gene expression, deregulation of miRNAs, metabolomic alterations, cellular migration and proliferation. Our results demonstrate that not only is the concentration of cfDNA higher in cancer patients, as previously shown by other studies^21,22^, but also that significant alterations can be found in the gene expression and phenotype of the cell lines, indicating a possible transformation of the cells by the tumor cfDNA. EMT is a widely cited process in metastasis progression in various types of cancer^23,24^, our findings also corroborated the relation with cancer progression and EMT.

The increase of *MMP9* and *CD44* have been the focus of many studies in the EMT process of compression and PCa progression. MMPs are part of the zinc-dependent protease family and play an important role in the proteolytic degradation of structural components of the extracellular matrix^25,26^. In PCa, *MMP2* and *MMP9*, in particular, have already been associated with metastasis, since high levels of both metalloproteases have been found in plasma^27,28^. The secretion of these proteins by cells can be a factor in the overexpression of the MMP9 gene, with no alteration of this protein in the cellular extract. In this context, the increase of *CD44*, a cellular adhesion molecule, has also been associated with neoplastic processes. The role of *CD44* in tumorigenesis is related to its connection with extracellular matrix components such as hyaluronic acid, osteopontin and growth factors present in the tumor microenvironment^29,30^.

We also demonstrated that besides the genetic alteration, the deregulation of miRNAs expression was triggered by the cfDNA treatments. The upregulation of miR-125b-5p in the treatment with T3/T4 cfDNA was associated with the malignant transformation in this cell, since miR-125b-5p is abnormally expressed in multiple cancers and is closely related to invasion and metastasis. In PCa, previous studies have shown an upregulation of miR-125b in malignant prostate cancer cell lines as well as clinical tissues of prostate cancer^31,32^. In other tumors the overexpression of the same miRNA promoted an increase in cellular migration, a significant decrease in expression of E-cadherin, and an increase in the expression of genes, such as MMP9 in PC-1 cells, characterizing the EMT in those cells^33^. These results corroborate our findings about the treatment with T3/T4 cfDNA, it also suggests that the miR-125b can be a biomarker for the detection of PCa.

In the treatment with T1/T2 cfDNA, cell lines showed a different expression pattern than T3 / T4. This result suggests that the tumorigenesis process can occur through different cellular responses, in different PCa stages and affects different types of cells in its own way. The up or downregulation of miRNAs after the treatment with T1/T2 cfDNA has been described in studies with oncoMIR or tumor suppressors^34–40^. Although, the action of such oncoMIRs in cancer regulation is still debatable, the diverse observed actions might be due to the tumor heterogeneity. This hypothesis is also reflected in our findings where the cfDNA isolated from different cancer stages affected non-tumor cells in distinct ways.

Analysis of tryptophan levels in the cells treated with cfDNA T3/T4 also suggests the triggering of a tumorigenesis process in the non-tumor cells. Tryptophan, a metabolite indicated in our analysis, is an amino acid that has already been reported in other studies on cancer cell metabolism. Tryptophan catabolism is a known mechanism involved in immune system modulation and is widely studied in cancer. The treatment of T3/T4 cfDNA promotes higher consumption of this amino acid by the cell. In the tumor microenvironment, depletion of tryptophan and its downstream metabolites, promotes the inhibition of T lymphocytes and natural killer, favoring tumor escape and proliferation^41,42^.

Our results demonstrate higher consumption of tryptophan by the RWPE-1 cells after the treatment with T3/T4 cfDNA, which can be further evidence of the tumorigenesis process. We understand that T3/T4 cfDNA and T1/T2 cfDNA can trigger different responses, indeed, each tumor stage possesses its own characteristics, which we believe can directly affect the profile of the cfDNA released and consequently its activity in the recipient cell. Evaluating together our findings on gene expression, miRNA regulation and tryptophan consumption, we understand that the increased cellular migration observed after the treatment with T3/T4 cfDNA is further evidence of the role of cfDNA in the malignant transformation of those cells.

Our hypothesis about how the cfDNA alters the biology of the recipient cells are based on other findings that are in accordance with our results^43,44^. Thus, we believe that cfDNA is a biologically active molecule that is released by cells continuously, which can enter healthy cells and integrate into their genome, causing changes in the DNA sequence, such as deletions, duplications and rearrangements^43,45^. In addition, alterations can be triggered by signaling pathways activated by the interaction of cfDNA with certain cell receptors or by increasing the transcriptional levels of certain genes in an interaction similar to what is observed with aptamers or antibodies^46^_._ Thus, it seems that cfDNA can significantly influence the functional activity of cells by activating signaling pathways and decreasing apoptosis levels^45^. However, we believe that tumor cfDNA could have different activities and interaction mechanisms depending on the cell from which it originates.

Further studies are warranted to characterize cfDNA (i.e. sequencing, copy number variation, epigenetic alterations) observed in each staging tumor, as well as the comparison of the role of the cfDNA isolated from groups of the same age to better understand the biological mechanisms of tumor cfDNA.

Overall, we have determined that tumor cfDNA besides being a potential biomarker candidate for cancer detection, exhibits biological activity, promoting tumor-like alterations on non-tumor cells. Therefore, by linking the events already demonstrated in other studies with the data obtained in the present work it would be restrictive to think that the progression of cancer is not related to cfDNA released by cancer cells.

## Methods

### Patients

The study was approved by the Ethics Committee of the Clinical Hospital, Federal University of Uberlandia, under ruling number 562.678. All research was performed in accordance with the relevant guidelines and regulations. Written informed consent was obtained from all the participants of this study. The median age of the 22 patients with prostate cancer was 62 years (range, 52–75 years, Table 1). The T1/T2 group patients did not receive preoperative antitumor therapy, whereas the T3/T4 group composed of patients in the metastatic phase had received hormone block therapies and radiotherapy. The median age of the 14 healthy male individuals was 29 years (range 28–44 years). These individuals were not diagnosed with any previous malignant diseases and were recruited as a control group.

**Table 1:** Gleason score and tumor TNM staging of prostate cancer patients.

Characteristics	Patients (N = 22)
**Age**	**Years**
Mean	61.26
Range	52–75
**Gleason Score**	**N (%)**
6 (3 + 3)	06 (27)
7 (3 + 4 and 4 + 3)	07 (32)
8 (4 + 4)	05 (23)
9 (4 + 5 and 5 + 4)	04 (18)
**TNM Staging**^24^	**N (%)**
T1	05 (23)
T2	10 (45)
T3	03 (14)
T4	04 (18)
**Treatment of patients in T3/T4**	Radiotherapy and hormone block

### Cell line culture

RWPE-1 and PNT-2 cell lines were cultured in keratinocyte medium supplemented with pituitary extract (0.05 mg/mL) and epidermal growth factor (EGF) (5 ng/mL), as well as RPMI medium supplemented with 10% fetal bovine serum, respectively. In addition to these supplements, 100 U/mL penicillin and 100 mg/mL streptomycin were added to the media. Both cell lines were cultivated at 37 ºC and 5% CO_2_ until reaching 90% confluence, when the cells were trypsinized and plated for later assays. During cultivation the media was changed every three days.

### cfDNA

#### Isolation

The cfDNA was extracted from 500 μL of plasma using XCF complete exosome and cfDNA isolation kit (System Biosciences), according to the manufacturer’s instructions. From the centrifuged plasma 500 μL were removed and mixed with 12 μL of reagent A, the mixture was vortexed for 10 s and incubated at 55 ºC for 10 min. Subsequently, 128 μL of ExoQuick reagent was combined into this mixture which was then vigorously vortexed and incubated for 30 min at 4 °C. After incubation the mixture was centrifuged at 13.000 × g for 10 min and 1 mL of binding buffer was added to the supernatant, this mixture was transferred to a column with a collecting tube and centrifuged for 3,300 × g for 2 min. The column was washed 2 times with a wash buffer and finally eluted with the respective elution buffer provided in the kit.

#### Characterization

The cfDNA size of each pool was analyzed by capillary electrophoresis (CE) using a High sensitivity DNA microchip kit, i.e., the Agilent 2100 Bioanalyzer (Agilent Technologies). The software automatically calculates the size of each fragment which is represented as an electropherogram. cfDNA quantification in the samples collected was performed by qPCR. Primer LINE was used: 5′-GGATTAAGAAAATGTGGCACCATATACACCATGG-3′ (*forward)*; 5′-ATAGTTTACTGAGAATGATGGTTTCCAATTTC-3′ (*reverse)*, and synthesized by Integrated DNA Technologies (IDT). PCR conditions were 95 °C for 10 min, followed by 40 cycles of 15 s denaturation at 95 °C, and 1 min annealing at 60°C^47^. The absolute concentration of the target gene was calculated using a standard curve. For this study the standard curve was generated using five serial dilutions of commercial genomic DNA (5000; 500; 50; 5 and 0.5 pg/μL). Each biological replicate was quantified in duplicate, and the standard curve quantified in triplicate for each run.

#### Incubation

The first prepared pool contained cfDNA obtained from the 14 healthy donors, used as controls (cfDNA Healthy), the second pool contained cfDNA from the 15 PCa patients staged at T1 and T2 (T1/T2 cfDNA), and the last prepared pool contained cfDNA of the seven PCa patients staged at T3 and T4 (T3/T4 cfDNA). Approximately 10 ng of cfDNA from each patient was used for pool preparation. RWPE-1 and PNT-2 cell lines were seeded in 6-well plates (2 × 10^3^ cells / well), treated with 20 ng of each cfDNA pool (cfDNA Healthy, T1/T2 or T3/T4) and incubated for 24 h at 37 °C and 5% CO_2_. Untreated cells were also evaluated and considered as a control for the experiments. The assay was conducted in triplicate. After cfDNA incubation, 100 μL of the supernatant of each replicate from both cell lines were collected for metabolite analysis. Subsequently, the cells were trypsinized for total RNA extraction.

### PCR

#### Analysis of gene expression and microRNA

For RT- qPCR analyses the total RNA was extracted from RWPE-1 and PNT-2 cell lines treated with cfDNA pools (cfDNA Healthy, T1/T2 or T3/T4) for 24 h. Extraction was performed by using mirVana kit (Thermo Fisher Scientific) according to the manufacturer's recommendations.

#### RT-qPCR

cDNA was synthesized from 1 μg of total RNA using the *M-MLV Reverse Transcriptase kit* according to the manufacturer's instructions (Thermo Fisher Scientific). Reverse transcriptase reaction was performed at 37 °C for 60 min using the Mycycler thermal cycler equipment (Thermo Fisher Scientific). qPCR reactions were performed using the 7300 Real Time PCR Systems apparatus (Applied Biosystems) to evaluate the expression of seven genes, vimentin *(VIM*), E-cadherin (*ECAD*), N-cadherin (*NCAD*), cell adhesion molecule epithelial (*EPCAM*), matrix metalloproteinase 9 (*MMP9*), cell surface glycoprotein (*CD44*), and transmembrane glycoprotein (*CD133*), all related to PCa progression and EMT. Cycle conditions were: 95 °C for 10 min, followed by 40 cycles at 95 °C for 15 s and 60 °C for 60 s. The endogenous gene used in this assay was beta-2-microglobulin (*B2M)*. The threshold fluorescence was manually configured for all plates using SDS software (Applied Biosystems). Analyses were performed from the cycle threshold (Ct) using DataAssist v3.01 software (Applied Biosystems).

#### microRNA expression analysis

The differential microRNA expression profile between patient-derived cfDNA-treated cell line (PNT-2) and healthy donor-derived cfDNA-treated cell line as well as untreated cell lines was assessed using the nCounter technology employing the nCounter Human v.3 miRNA Expression assay (NanoString Technologies) at the Molecular Oncology Research Center of Barretos Cancer Hospital, as previously described^48^. nCounter Human v.3 miRNA Expression assay contains 800 miRNAs including clinically relevant human miRNAs in addition to 6 positive and 8 negative controls, 3 positive ligation and 3 negative ligation controls, 5 mRNA reference genes (for mRNA contamination check) and 5 spike-ins. Expression assay was performed in biological triplicate, for all groups.

All procedures regarding sample preparation, hybridization, detection and scanning were performed according to the manufacturer’s instructions (NanoString Technologies). Briefly, a total amount of 100 ng RNA was incubated with tags for "bridge" formation followed by ligation and purification steps. Then, pre-prepared samples were hybridized with hybridization buffer, reporter probe and capture probe and then, incubated at 65 °C for 24 h. Subsequently, samples were loaded to the nCounter PrepStation (NanoString Technologies), which automatically performs purification steps and cartridge preparation. Finally, the cartridges containing immobilized and aligned reporter complexes were transferred to the nCounter Digital Analyzer (NanoString Technologies), set at a high-resolution setting, which captures up to 555 fields of view (FOVs) per sample providing all gene counts. Reporter probe counts were captured by the nCounter Digital Analyzer and raw data was collected and pre-processed by nSolver Analysis Software v4.0 (NanoString Technologies).

The nine samples presented acceptable quality control parameters and were evaluated in further steps. Normalization and differential expression were performed in the R statistical environment (v3.6.2). Expression levels were first accessed with quantro package^49^ (v1.18.0), an R package that applies a data-driven approach in order to lead the selection of the normalization method. A filter was applied to remove from subsequent analysis all miRNA for which the expression for all samples remained below the mean plus ten standard deviations of negative control counts. Normalization was performed with the NanoStringNorm package^50^ (v1.2.1), using the geometric mean option of positive controls, followed by the geometric mean option for the 10 miRNAs with the lowest coefficient of variation in the panel. After normalization, data were log2 transformed and differential expression was performed using one-way ANOVA and pairwise t-test with FDR correction for multiple comparisons with ggpubr package (v0.2.2).

#### Protein extraction and quantification

Cells were washed with PBS and disrupted in lyses buffer (20 mM Tris–HCl (pH 7.5), 150 mM NaCl, 1 mM Na_2_EDTA, 1 mM EGTA, 1% Triton X-100, 2.5 mM sodium pyrophosphate, 1 mM β-glycerophosphate, 1 mM Na_3_VO_4_ and1 µg/ml leupeptin). After three sonication cycles at 45 W for 5 min each (samples were kept on ice between sonication cycles) in a sonicator bath of 800 mL (Unique), the samples were centrifuged at 20, 000 × g for 30 min at 4 °C. The protein concentration was determined by the Bradford method (Bio-Rad).

#### Western blotting

Proteins were submitted to SDS–PAGE and electrotransferred to PVDF membranes (GE Lifesciences). The membranes were blocked with wash buffer (25 mM Tris–HCl, pH 7.5, 0.5 M NaCl and 0.1% Tween-20) containing 5% non-fat dry milk and incubated with a primary antibody following manufacturer’s instructions. Mouse anti-CD44 (#3750), rabbit anti-MMP-9 (#2270) and rabbit anti-GAPDH (#2118) were purchased from Cell Signaling. After 1 h of incubation with horseradish peroxidase-conjugated goat anti-rabbit IgG (#7074, Cell Signaling) or horse anti-mouse IgG (#7076, Cell Signaling) secondary antibodies, the antibody-protein complex was detected using ECL Western Blotting Detection Reagents (GE Lifesciences) using a CCD-Camera (Image QuantLAS 4000 mini).

#### Migration assay

RWPE-1 and PNT-2 cells (2 × 10^4^) were seeded and cultured in a 12-well plate until 80% of confluence. A 200 ul pipette was used to scratch the cells and 20 μg of each cfDNA (cfDNA Healthy, T1/T2 or T3/T4) were added to the cultures. Cell migration was monitored by microscopy and images were taken at 0 and 24 h. Area of migration was quantified using the software Fiji-Image J (NIH, Bethesda, MD). Experiments were performed in triplicate.

#### Proliferation assay

RWPE-1 and PNT-2 cells (2 × 10^3^) were seeded in a 96-well plate and treated with 20 μg of each cfDNA (cfDNA Healthy, T1/T2 or T3/T4) for 24 h at 37 °C and 5% CO_2_. CytoSelect Cell Proliferation Assay Reagent (CELL BIOLABS) was used according to the manufacturer's instructions to evaluate the cell proliferation. The assay was performed in triplicate.

### Metabolomic

Supernatant from RWPE-1 and PNT-2 cells incubated with cfDNA was taken for metabolite profiling. The percentage of changed metabolites of the cfDNA-induced metabolome analysis was compared to control. After, the supernatant was incubated with 1 mL of spectroscopic grade methanol at -80 °C for 4 h and centrifuged for 15 min at 13,000 × g. The supernatant was transferred to another microtube to concentrate the sample in a vacuum concentrator for 30 min and subsequently the samples were lyophilized. For mass spectrometry analyses the samples were resuspended in 500 µL of spectroscopic grade methanol, and filtered through a 0.22 µm pore-tip syringe filter for MS analysis. The assay was performed in triplicate.

#### IES/MS

Direct infusion IES/MS (Electrospray Ionization Mass Spectrometry) analyzes were performed by a liquid chromatography (Agilent Technologies model Infinity 1260) from sample injection coupled with a Q-TOF high-resolution mass spectrometer (Agilent Technologies Model 6520 B QTOF). The chromatographic parameters were: 0.2 mL/min mobile phase constant flow with 90% methanol composition and 10% formic acid acidified water [0.1% (vv^–1^)], the volume of sample injection was 2 µL. The ionization parameters were: nebulizer pressure of 20 psi, drying gas at 8 L/min at 220ºC and a capillary energy of 4.5 kVA. The acquisition mode MS1 had an *m/z* range of 100 to 3000, an acquisition rate of 1 spectra/sec with the accumulation of 1000 MS/spectra. The scan source parameters of Fragmentor and Skimmer were 175 and 65 V, respectively. Data were acquired in positive and negative modes. Injection of each sample were performed in triplicate.

#### Identification

A high-Resolution Mass Spectrometer (Agilent Technologies Model 6520 B QTOF) with an electrospray ionization source was used for a further injection of samples to identify the most significant compounds from the chemometric analysis. Thus, the samples were solubilized in methanol/water (4:1) and introduced using a 100 μL syringe, adapted to a 200 μL/h direct flow infusion pump. Ionization was performed with nebulizing gas at 20 psi, the drying gas heated to 200 °C with a flow rate of 8.0 L/min and 4.5 kV was applied to the capillary. The product-ion spectra were acquired at six different collision energy levels (5, 10, 15, 20, 25, and 30 eV). Data were acquired in positive and negative modes. The compound identification was performed considering the high-resolution mass (the error with the exact mass less than 10 ppm), and the mass spectra/mass spectra (MS/MS). These data were cross-checked with databases (MassBank Europe, Human Metabolome Database, Metabolomics Workbench, MassBank of North America and METLIN).

### Statistical analysis

Statistical analysis was performed using GraphPad Prism software (version 7.0). cfDNA concentration data were analyzed using test t student and Mann Whitney test. The qPCR, migration and cell proliferation data were compared using non-parametric tests (Kruskal–Wallis test followed by Dunn's post-test). Comparisons were considered statistically significant when *p*-value was less than 0.05 (p < 0.05).

## Supplementary information

Supplementary information.

## Data Availability

The data generated and/or analyzed during the current study will be made available from the corresponding author upon reasonable request.

## References

[1] Mandel & Metais (1948). Les acides nucleiques du plasma sanguin chez l’homme. CR Acad. Sci..

[2] Volik S, Alcaide M, Morin RD, Collins C (2016). Cell-free DNA (cfDNA): clinical significance and utility in cancer shaped by emerging technologies. Mol. Cancer Res..

[3] Riva F (2016). Clinical applications of circulating tumor DNA and circulating tumor cells in pancreatic cancer. Mol. Oncol..

[4] Crowley E, Di Nicolantonio F, Loupakis F, Bardelli A (2013). Liquid biopsy: monitoring cancer-genetics in the blood. Nat. Rev. Clin. Oncol..

[5] Qin Z, Ljubimov VA, Zhou C, Tong Y, Liang J (2016). Cell-free circulating tumor DNA in cancer. Chin. J. Cancer.

[6] Gormally E, Caboux E, Vineis P, Hainaut P (2007). Circulating free DNA in plasma or serum as biomarker of carcinogenesis: Practical aspects and biological significance. Mutat. Res. Rev. Mutat. Res..

[7] Thierry AR (2014). Clinical validation of the detection of KRAS and BRAF mutations from circulating tumor DNA. Nat. Med..

[8] Zinkova A, Brynychova I, Svacina A, Jirkovska M, Korabecna M (2017). Cell-free DNA from human plasma and serum differs in content of telomeric sequences and its ability to promote immune response. Sci. Rep..

[9] Garcia-Olmo DC, Garcia-Olmo D (2012). Biological role of cell-free nucleic acids in cancer: the theory of genometastasis. Crit. Rev. Oncog..

[10] García-Olmo DC (2010). Cell-free nucleic acids circulating in the plasma of colorectal cancer patients induce the oncogenic transformation of susceptible cultured cells. Cancer Res..

[11] Bronkhorst AJ (2016). Characterization of the cell-free DNA released by cultured cancer cells. Biochim. Biophys. Acta Mol. Cell Res..

[12] Bennett CW, Berchem G, Kim YJ, El-Khoury V (2016). Cell-free DNA and next-generation sequencing in the service of personalized medicine for lung cancer. Oncotarget.

[13] Brabletz T, Kalluri R, Nieto MA, Weinberg RA (2018). EMT in cancer. Nat. Rev. Cancer.

[14] Heerboth S (2015). EMT and tumor metastasis. Clin. Transl. Med..

[15] García-Olmo D, García-Olmo DC (2001). Functionality of circulating DNA: the hypothesis of genometastasis. Ann. N. Y. Acad. Sci..

[16] Takai E (2015). Clinical utility of circulating tumor DNA for molecular assessment in pancreatic cancer. Sci. Rep..

[17] Wang M (2017). Role of tumor microenvironment in tumorigenesis. J. Cancer.

[18] Bergers G, Benjamin LE (2003). Tumorigenesis and the angiogenic switch. Nat. Rev. Cancer.

[19] Tabassum DP, Polyak K (2015). Tumorigenesis: it takes a village. Nat. Rev. Cancer.

[20] McCaffrey LM, Macara IG (2011). Epithelial organization, cell polarity and tumorigenesis. Trends Cell Biol..

[21] Valpione S (2018). Plasma total cell-free DNA (cfDNA) is a surrogate biomarker for tumour burden and a prognostic biomarker for survival in metastatic melanoma patients. Eur. J. Cancer.

[22] Hyun MH (2017). Quantification of circulating cell-free DNA to predict patient survival in non-small-cell lung cancer. Oncotarget.

[23] Brabletz T, Kalluri R, Nieto MA, Weinberg RA (2018). EMT in cancer. Nat. Rev. Cancer.

[24] Savagner P (2010). The epithelial-mesenchymal transition (EMT) phenomenon. Ann. Oncol..

[25] Manfredi MA (2008). Increased incidence of urinary matrix metalloproteinases as predictors of disease in pediatric patients with inflammatory bowel disease. Inflamm. Bowel Dis..

[26] Kessenbrock K, Plaks V, Werb Z (2010). Matrix metalloproteinases: regulators of the tumor microenvironment. Cell.

[27] Moroz A (2013). Finasteride inhibits human prostate cancer cell invasion through MMP2 and MMP9 downregulation. PLoS ONE.

[28] Aalinkeel R (2011). Overexpression of MMP-9 contributes to invasiveness of prostate cancer cell line LNCaP. Immunol. Invest..

[29] Ponta H, Sherman L, Herrlich PA (2003). CD44: from adhesion molecules to signalling regulators. Nat. Rev. Mol. Cell Biol..

[30] Naor D, Nedvetzki S, Golan I, Melnik L, Faitelson Y (2002). CD44 in Cancer. Crit. Rev. Clin. Lab. Sci..

[31] Banzhaf-Strathmann J, Edbauer D (2014). Good guy or bad guy: the opposing roles of microRNA 125b in cancer. Cell Commun. Signal..

[32] Mullane SA (2015). miR125 and miR200a as potential circulating miRNA biomarkers in metastatic urothelial carcinoma patients treated with docetaxel. J. Clin. Oncol..

[33] Wu M (2020). Role of exosomal microRNA-125b-5p in conferring the metastatic phenotype among pancreatic cancer cells with different potential of metastasis. Life Sci..

[34] Bonci D (2008). The miR-15a–miR-16-1 cluster controls prostate cancer by targeting multiple oncogenic activities. Nat. Med..

[35] Li W (2015). miRNA-99b-5p suppresses liver metastasis of colorectal cancer by down-regulating mTOR. Oncotarget.

[36] Guo J, Gong G, Zhang B (2018). miR-539 acts as a tumor suppressor by targeting epidermal growth factor receptor in breast cancer. Sci. Rep..

[37] Nishida N (2011). MicroRNA-125a-5p is an independent prognostic factor in gastric cancer and inhibits the proliferation of human gastric cancer cells in combination with Trastuzumab. Clin. Cancer Res..

[38] Cimmino A (2005). miR-15 and miR-16 induce apoptosis by targeting BCL2. Proc. Natl. Acad. Sci..

[39] Jiang L (2010). Hsa-miR-125a-3p and hsa-miR-125a-5p are downregulated in non-small cell lung cancer and have inverse effects on invasion and migration of lung cancer cells. BMC Cancer.

[40] Svoronos AA, Engelman DM, Slack FJ (2016). OncomiR or tumor suppressor? The duplicity of MicroRNAs in cancer. Cancer Res..

[41] Prendergast GC (2011). Cancer: why tumours eat tryptophan. Nature.

[42] Platten M, Wick W, Van Den Eynde BJ (2012). Tryptophan catabolism in cancer: beyond IDO and tryptophan depletion. Can. Res..

[43] Mittra I (2015). Circulating nucleic acids damage DNA of healthy cells by integrating into their genomes. J. Biosci..

[44] Mittra I (2015). Circulating nucleic acids: a new class of physiological mobile genetic elements. F1000Research.

[45] Kostyuk SV (2012). Fragments of cell-free DNA increase transcription in human mesenchymal stem cells, activate TLR-dependent signal pathway, and suppress apoptosis. Biochem. Suppl. Ser. B Biomed. Chem..

[46] Zhou J, Rossi J (2017). Aptamers as targeted therapeutics: current potential and challenges. Nat. Rev. Drug Discov..

[47] Wu X, Tanaka H (2015). Aberrant reduction of telomere repetitive sequences in plasma cell-free DNA for early breast cancer detection. Oncotarget.

[48] Souza KCB (2019). Identification of cell-free circulating microRNAs for the detection of early breast cancer and molecular subtyping. J. Oncol..

[49] Hicks SC, Irizarry RA (2015). Quantro: a data-driven approach to guide the choice of an appropriate normalization method. Genome Biol..

[50] Waggott D (2012). NanoStringNorm: an extensible R package for the pre-processing of nanostring mRNA and miRNA data. Bioinformatics.

