# Supplementary material for: Cell-free DNA promotes malignant transformation in non-tumor cells
Source: Sci Rep. 2020 Dec 10;10:21674. doi: 10.1038/s41598-020-78766-5 (PMC7728762; doi:10.1038/s41598-020-78766-5)
Supplement: Supplementary file 1 — Supplementary information. [file 41598_2020_78766_MOESM1_ESM.docx]

**Supplementary Information**

**Cell-free DNA promotes malignant transformation in non-tumor cells**

**Aline Gomes Souza^a, *^, Victor Alexandre F. Bastos^a^, Patricia Tieme Fujimura^a^, Izabella Cristina C. Ferreira^a^, Letícia Ferro Leal^b^, Luciane Sussuchi da Silva^b^, Ana Carolina Laus^b^, Rui Manuel Reis ^b,d,e^, Mario Machado Martins^a^, Paula Souza Santos^a^, Natássia C. Resende Corrêa^a^, Karina Marangoni^a^, Carolina Hassibe Thomé ^f^_,_ Leandro Machado Colli ^g^, Luiz Ricardo Goulart^a,c^, and Vivian Alonso Goulart^a^.**

^a^Laboratory of Nanobiotechnology, Institute of Biotechnology, Federal University of Uberlândia, Uberlândia/MG, 38400-902, Brazil.

^b^Molecular Oncology Research Center, Barretos Cancer Hospital, Barretos -SP, 14784-400, Brazil.

^c^University of California-Davis, Dept. of Medical Microbiology and Immunology, Davis/CA, 95616, USA.

^d^Life and Health Sciences Research Institute (ICVS), Medical School, University of Minho, Braga, Portugal;

^e^3ICVS/3B’s-PT Government Associate Laboratory, Braga, Portugal;

* Corresponding author: [alingosouza@yahoo.com.br](mailto:alingosouza@yahoo.com.br)


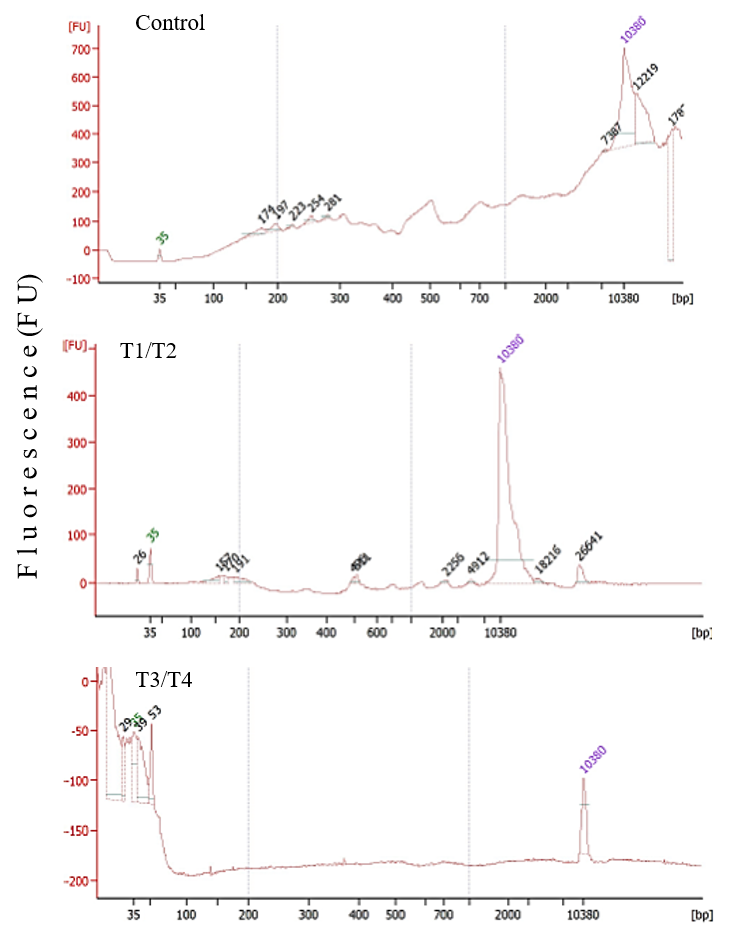


**Supplementary Figure S1**. Capillary Electrophoresis of plasma cfDNA from prostate cancer patients. Marker cfDNA of 35 and 10380 bp is present in the plasma patients. Differences are demonstrated between control and cancer patients in bp regions corresponding to the markers and in the range 100-300 bp. Data are reported for pools of plasma from the number of patients listed in Table 1.

**A**

**B**

**Supplementary Figure S2. Quantification of concentration of cfDNA in healthy individuals (A)** and patients with tumor (**B**). Data are expressed as the mean ± SD.


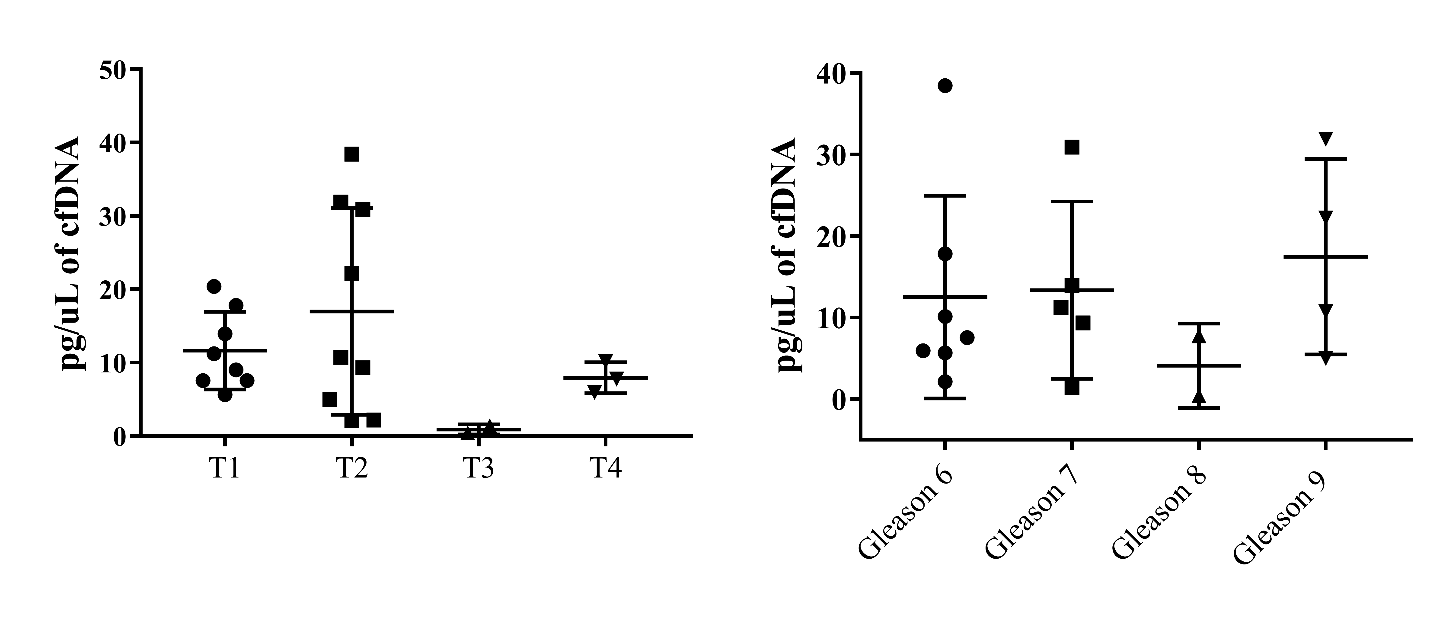


**A**

**B**

**Supplementary Figure S3.** Comparison of the concentration of cfDNA among the staging **(A)** and Gleason score **(B).** Data are expressed as the mean ± SD. No statistical difference was observed (p < 0.05; Mann–Whitney *U*-test).

.


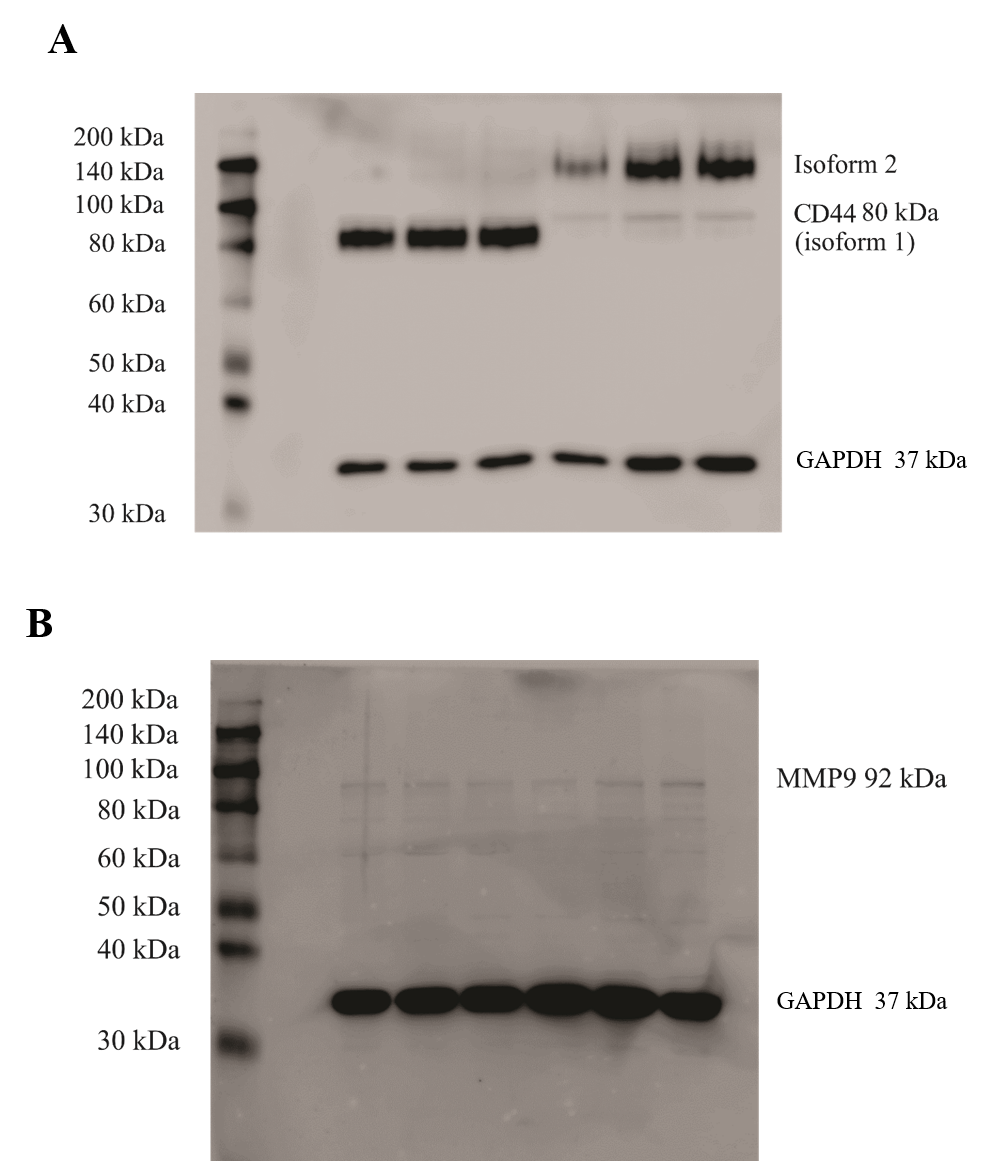


**Supplementary Figure S4:**  Levels of CD44 isoforms and MMP9 in RWPE-1 and PNT-2 were analyzed by western blotting. GAPDH was used as loading control.


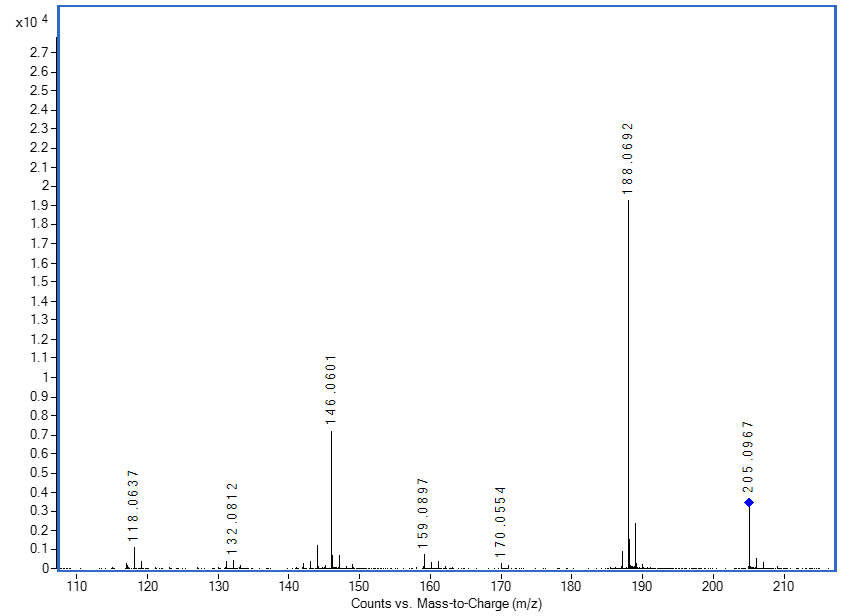


**Supplementary Figure S5.** Precursor ions and fragment ions of tryptophan-mass spectrometry parameters.

**Supplementary Table S1.** Statistical analysis of miRNAs up or downregulated in treatment with tumor cfDNA compared to control groups

| .y. | group1 | group2 | p.adj | log2fc |
| --- | --- | --- | --- | --- |
| hsa-let-7f-5p | Normal | T1 | 0.022 | 1.058333 |
| hsa-let-7f-5p | T1 | T3 | 0.022 | -1.05867 |
| hsa-miR-125a-5p | Normal | T1 | 0.009 | 1 |
| hsa-miR-125a-5p | T1 | T3 | 0.0051 | -1.03367 |
| hsa-miR-125b-5p | Normal | T3 | 0.0031 | -0.70367 |
| hsa-miR-125b-5p | T1 | T3 | 0.0031 | -0.71233 |
| hsa-miR-16-5p | Normal | T1 | 0.0017 | 0.923333 |
| hsa-miR-16-5p | T1 | T3 | 0.001 | -1.17667 |
| hsa-miR-27b-3p | Normal | T1 | 0.00081 | 1.007 |
| hsa-miR-27b-3p | T1 | T3 | 0.0065 | -0.84667 |
| hsa-miR-32-5p | Normal | T1 | 0.00026 | 0.641333 |
| hsa-miR-32-5p | T1 | T3 | 0.025 | -0.64267 |
| hsa-miR-493-3p | Normal | T1 | 0.027 | -1.46433 |
| hsa-miR-493-3p | T1 | T3 | 0.04 | 1.107 |
| hsa-miR-539-5p | Normal | T1 | 0.018 | -1.72433 |
| hsa-miR-539-5p | T1 | T3 | 0.018 | 1.087667 |
| hsa-miR-99b-5p | Normal | T1 | 0.015 | 1.654667 |
| hsa-miR-99b-5p | T1 | T3 | 0.0085 | -1.98033 |

**Supplementary Table S2.** Statistical analysis of miRNAs between control group (cells without treatment) and healthy cfDNA.

| miRNA | P_Control | Q_Control |
| --- | --- | --- |
| hsa-let-7b-5p | 0.42 | 0.87 |
| hsa-let-7c-5p | 0.94 | 0.96 |
| hsa-let-7d-5p | 0.4 | 0.87 |
| hsa-let-7e-5p | 0.92 | 0.96 |
| hsa-let-7f-5p | 0.03 | 0.86 |
| hsa-let-7g-5p | 0.68 | 0.93 |
| hsa-let-7i-5p | 0.42 | 0.87 |
| hsa-miR-100-5p | 0.21 | 0.86 |
| hsa-miR-106a-5p | 0.94 | 0.96 |
| hsa-miR-106b-5p | 0.22 | 0.86 |
| hsa-miR-107 | 0.34 | 0.86 |
| hsa-miR-10a-5p | 0.66 | 0.93 |
| hsa-miR-1180-3p | 0.17 | 0.86 |
| hsa-miR-1246 | 0.07 | 0.86 |
| hsa-miR-125a-5p | 0.59 | 0.93 |
| hsa-miR-125b-5p | 0.34 | 0.86 |
| hsa-miR-126-3p | 0.84 | 0.95 |
| hsa-miR-1260a | 0.66 | 0.93 |
| hsa-miR-1306-5p | 0.5 | 0.93 |
| hsa-miR-130a-3p | 0.12 | 0.86 |
| hsa-miR-130b-3p | 0.29 | 0.86 |
| hsa-miR-132-3p | 0.91 | 0.95 |
| hsa-miR-135b-5p | 0.3 | 0.86 |
| hsa-miR-137 | 0.9 | 0.95 |
| hsa-miR-140-5p | 0.34 | 0.86 |
| hsa-miR-141-3p | 0.15 | 0.86 |
| hsa-miR-148a-3p | 0.78 | 0.95 |
| hsa-miR-148b-3p | 0.63 | 0.93 |
| hsa-miR-149-5p | 0.14 | 0.86 |
| hsa-miR-151a-3p | 0.17 | 0.86 |
| hsa-miR-151a-5p | 0.76 | 0.94 |
| hsa-miR-155-5p | 0.25 | 0.86 |
| hsa-miR-15a-5p | 0.27 | 0.86 |
| hsa-miR-15b-5p | 0.8 | 0.95 |
| hsa-miR-16-5p | 0.95 | 0.96 |
| hsa-miR-181a-2-3p | 0.87 | 0.95 |
| hsa-miR-181a-3p | 0.097 | 0.86 |
| hsa-miR-181a-5p | 0.31 | 0.86 |
| hsa-miR-181d-5p | 0.15 | 0.86 |
| hsa-miR-181c-5p | 0.71 | 0.93 |
| hsa-miR-182-5p | 0.54 | 0.93 |
| hsa-miR-183-5p | 0.015 | 0.6 |
| hsa-miR-185-5p | 0.48 | 0.93 |
| hsa-miR-186-5p | 0.0093 | 0.6 |
| hsa-miR-18a-5p | 0.62 | 0.93 |
| hsa-miR-193b-3p | 0.89 | 0.95 |
| hsa-miR-196a-5p | 0.53 | 0.93 |
| hsa-miR-196b-5p | 0.58 | 0.93 |
| hsa-miR-197-3p | 0.73 | 0.93 |
| hsa-miR-19a-3p | 0.43 | 0.87 |
| hsa-miR-19b-3p | 0.43 | 0.87 |
| hsa-miR-200a-3p | 0.72 | 0.93 |
| hsa-miR-200b-3p | 0.22 | 0.86 |
| hsa-miR-200c-3p | 0.23 | 0.86 |
| hsa-miR-203a-3p | 0.31 | 0.86 |
| hsa-miR-205-5p | 0.25 | 0.86 |
| hsa-miR-21-5p | 0.18 | 0.86 |
| hsa-miR-210-3p | 0.65 | 0.93 |
| hsa-miR-22-3p | 0.13 | 0.86 |
| hsa-miR-221-3p | 0.78 | 0.95 |
| hsa-miR-221-5p | 0.37 | 0.87 |
| hsa-miR-222-3p | 0.28 | 0.86 |
| hsa-miR-23a-3p | 0.4 | 0.87 |
| hsa-miR-23b-3p | 0.52 | 0.93 |
| hsa-miR-24-3p | 0.84 | 0.95 |
| hsa-miR-25-3p | 0.19 | 0.86 |
| hsa-miR-26a-5p | 0.32 | 0.86 |
| hsa-miR-26b-5p | 0.26 | 0.86 |
| hsa-miR-27b-3p | 0.31 | 0.86 |
| hsa-miR-28-3p | 0.73 | 0.93 |
| hsa-miR-28-5p | 0.66 | 0.93 |
| hsa-miR-296-5p | 0.51 | 0.93 |
| hsa-miR-29a-3p | 0.81 | 0.95 |
| hsa-miR-29b-3p | 0.18 | 0.86 |
| hsa-miR-29c-3p | 0.82 | 0.95 |
| hsa-miR-301a-3p | 1 | 1 |
| hsa-miR-30a-3p | 0.039 | 0.86 |
| hsa-miR-30a-5p | 0.6 | 0.93 |
| hsa-miR-30b-5p | 0.42 | 0.87 |
| hsa-miR-30c-5p | 0.88 | 0.95 |
| hsa-miR-30d-5p | 0.16 | 0.86 |
| hsa-miR-30e-3p | 0.68 | 0.93 |
| hsa-miR-30e-5p | 0.72 | 0.93 |
| hsa-miR-31-5p | 0.7 | 0.93 |
| hsa-miR-3161 | 0.62 | 0.93 |
| hsa-miR-32-5p | 0.15 | 0.86 |
| hsa-miR-320e | 0.34 | 0.86 |
| hsa-miR-324-5p | 0.91 | 0.95 |
| hsa-miR-331-3p | 0.82 | 0.95 |
| hsa-miR-33a-5p | 0.27 | 0.86 |
| hsa-miR-342-3p | 0.86 | 0.95 |
| hsa-miR-345-5p | 0.28 | 0.86 |
| hsa-miR-34a-5p | 0.9 | 0.95 |
| hsa-miR-361-3p | 0.27 | 0.86 |
| hsa-miR-361-5p | 0.042 | 0.86 |
| hsa-miR-362-3p | 0.74 | 0.93 |
| hsa-miR-362-5p | 0.37 | 0.87 |
| hsa-miR-365a-3p | 0.64 | 0.93 |
| hsa-miR-374a-5p | 0.85 | 0.95 |
| hsa-miR-374b-5p | 0.14 | 0.86 |
| hsa-miR-374c-5p | 0.57 | 0.93 |
| hsa-miR-378i | 0.69 | 0.93 |
| hsa-miR-421 | 0.073 | 0.86 |
| hsa-miR-423-3p | 0.098 | 0.86 |
| hsa-miR-423-5p | 0.23 | 0.86 |
| hsa-miR-424-5p | 0.8 | 0.95 |
| hsa-miR-425-5p | 0.86 | 0.95 |
| hsa-miR-4286 | 0.16 | 0.86 |
| hsa-miR-429 | 0.16 | 0.86 |
| hsa-miR-4443 | 0.73 | 0.93 |
| hsa-miR-450a-5p | 0.54 | 0.93 |
| hsa-miR-4521 | 0.061 | 0.86 |
| hsa-miR-454-3p | 0.19 | 0.86 |
| hsa-miR-493-3p | 0.41 | 0.87 |
| hsa-miR-503-5p | 0.82 | 0.95 |
| hsa-miR-539-5p | 0.55 | 0.93 |
| hsa-miR-551a | 0.39 | 0.87 |
| hsa-miR-574-3p | 0.24 | 0.86 |
| hsa-miR-574-5p | 0.56 | 0.93 |
| hsa-miR-579-3p | 0.048 | 0.86 |
| hsa-miR-582-5p | 0.89 | 0.95 |
| hsa-miR-590-5p | 0.64 | 0.93 |
| hsa-miR-612 | 0.71 | 0.93 |
| hsa-miR-651-5p | 0.093 | 0.86 |
| hsa-miR-660-5p | 0.56 | 0.93 |
| hsa-miR-6724-5p | 0.72 | 0.93 |
| hsa-miR-7-5p | 0.07 | 0.86 |
| hsa-miR-769-5p | 0.88 | 0.95 |
| hsa-miR-874-5p | 0.37 | 0.87 |
| hsa-miR-92a-3p | 0.95 | 0.96 |
| hsa-miR-93-5p | 0.43 | 0.87 |
| hsa-miR-934 | 0.59 | 0.93 |
| hsa-miR-96-5p | 0.59 | 0.93 |
| hsa-miR-98-5p | 0.061 | 0.86 |
| hsa-miR-99b-5p | 0.35 | 0.87 |

**Supplementary Table S3**. Mean of abundance levels of ions detected by metabolomic analysis in RWPE-1 treated with cfDNA healthy, cfDNA T1 / T2, cfDNA T3 / T4.

|  | Ions | Control | cfDNA Healthy | cfDNA T1/T2 | cfDNA T3/T4 |
| --- | --- | --- | --- | --- | --- |
| 1 | 104.107 | 11.080 | 13.940 | 13.220 | 15.240 |
| 2 | 110.072 | 2.650 | 2.680 | 2.710 | 2.740 |
| 3 | 114.984 | 6.920 | 7.410 | 8.030 | 8.080 |
| 4 | 116.070 | 7.040 | 6.980 | 7.000 | 6.840 |
| 5 | 118.087 | 3.040 | 3.040 | 3.020 | 2.950 |
| 6 | 130.050 | 22.650 | 22.780 | 22.820 | 22.620 |
| 7 | 130.093 | 3.820 | 3.920 | 4.130 | 3.780 |
| 8 | 132.102 | 13.880 | 13.850 | 13.760 | 13.470 |
| 9 | 136.062 | 7.170 | 7.150 | 7.300 | 7.130 |
| 10 | 143.001 | 5.590 | 5.640 | 5.860 | 5.660 |
| 11 | 147.077 | 8.170 | 8.200 | 8.140 | 8.030 |
| 12 | 147.112 | 2.800 | 2.780 | 2.770 | 2.720 |
| 13 | 148.945 | 3.110 | 3.180 | 3.450 | 3.600 |
| 14 | 152.032 | 6.490 | 6.190 | 6.340 | 6.310 |
| 15 | 154.084 | 2.900 | 2.720 | 2.820 | 2.750 |
| 16 | 156.076 | 4.530 | 4.630 | 4.610 | 4.430 |
| 17 | 157.083 | 5.040 | 4.900 | 3.020 | 3.530 |
| 18 | 158.973 | 12.970 | 13.990 | 15.110 | 15.220 |
| 19 | 169.058 | 11.860 | 11.230 | 11.500 | 11.370 |
| 20 | 174.014 | 3.480 | 3.160 | 3.340 | 3.390 |
| 21 | 175.119 | 96.870 | 95.980 | 97.050 | 92.640 |
| 22 | 176.066 | 3.860 | 3.470 | 3.680 | 3.700 |
| 23 | 176.121 | 7.490 | 7.340 | 7.450 | 7.140 |
| 24 | 183.078 | 0.000 | 2.500 | 0.000 | 0.000 |
| 25 | 185.115 | 10.600 | 10.440 | 9.910 | 8.430 |
| 26 | 191.040 | 13.580 | 12.010 | 12.750 | 13.090 |
| 27 | 197.101 | 8.210 | 7.350 | 7.810 | 7.750 |
| 28 | 204.056 | 17.280 | 21.090 | 16.110 | 12.040 |
| 29 | 205.060 | 40.020 | 50.150 | 20.120 | 12.040 |
| 30 | 216.923 | 2.930 | 2.510 | 2.500 | 2.590 |
| 31 | 217.104 | 16.850 | 15.640 | 16.720 | 17.030 |
| 32 | 219.083 | 4.060 | 3.470 | 3.770 | 3.810 |
| 33 | 239.106 | 96.800 | 98.150 | 99.070 | 97.970 |
| 34 | 240.109 | 9.540 | 9.640 | 9.750 | 9.670 |
| 35 | 241.103 | 4.820 | 4.870 | 4.920 | 4.860 |
| 36 | 261.088 | 89.890 | 87.270 | 89.680 | 87.720 |
| 37 | 261.130 | 29.230 | 27.420 | 29.140 | 29.710 |
| 38 | 262.091 | 8.870 | 8.600 | 8.860 | 8.720 |
| 39 | 262.134 | 3.300 | 3.060 | 3.270 | 3.330 |
| 40 | 263.085 | 4.540 | 4.370 | 4.510 | 4.420 |
| 41 | 277.061 | 6.700 | 6.870 | 6.770 | 6.590 |
| 42 | 283.070 | 52.340 | 47.760 | 50.510 | 50.440 |
| 43 | 284.073 | 5.250 | 4.830 | 5.070 | 5.080 |
| 44 | 285.067 | 2.660 | 2.510 | 2.580 | 2.580 |
| 45 | 293.025 | 2.720 | 2.940 | 3.030 | 3.170 |
| 46 | 299.044 | 5.600 | 5.380 | 5.450 | 5.320 |
| 47 | 301.141 | 7.660 | 8.540 | 6.910 | 5.630 |
| 48 | 305.157 | 14.590 | 13.840 | 14.580 | 14.950 |
| 49 | 337.172 | 7.030 | 7.550 | 7.780 | 7.430 |
| 50 | 341.029 | 6.490 | 5.680 | 6.130 | 6.230 |
| 51 | 343.026 | 2.470 | 2.560 | 2.380 | 2.470 |
| 52 | 349.183 | 6.070 | 5.650 | 6.030 | 6.240 |
| 53 | 351.058 | 2.600 | 2.240 | 2.390 | 2.590 |
| 54 | 357.088 | 6.290 | 6.040 | 6.160 | 5.900 |
| 55 | 362.242 | 4.150 | 3.780 | 3.430 | 3.080 |
| 56 | 379.282 | 2.430 | 2.670 | 2.630 | 2.620 |
| 57 | 383.117 | 4.640 | 4.080 | 4.340 | 4.300 |
| 58 | 385.176 | 2.290 | 2.580 | 2.470 | 2.170 |
| 59 | 393.209 | 2.330 | 0.000 | 0.000 | 0.000 |
| 60 | 398.242 | 39.010 | 43.740 | 47.990 | 45.280 |
| 61 | 398.988 | 3.430 | 2.870 | 3.150 | 3.260 |
| 62 | 399.245 | 9.110 | 10.140 | 11.110 | 10.490 |
| 63 | 400.985 | 2.450 | 2.520 | 2.980 | 0.000 |
| 64 | 407.158 | 2.890 | 3.000 | 0.000 | 2.880 |
| 65 | 413.218 | 9.330 | 10.590 | 10.130 | 9.200 |
| 66 | 413.267 | 2.280 | 2.410 | 2.670 | 2.630 |
| 67 | 414.221 | 3.290 | 3.790 | 3.830 | 3.460 |
| 68 | 418.784 | 6.220 | 4.520 | 4.720 | 4.730 |
| 69 | 419.285 | 3.170 | 2.660 | 2.560 | 2.790 |
| 70 | 429.140 | 2.450 | 2.350 | 2.470 | 2.380 |
| 71 | 435.200 | 3.080 | 3.020 | 3.130 | 2.990 |
| 72 | 437.194 | 34.780 | 50.380 | 42.480 | 58.760 |
| 73 | 438.197 | 8.860 | 12.640 | 19.390 | 14.750 |
| 74 | 439.203 | 2.540 | 3.150 | 2.660 | 3.080 |
| 75 | 451.122 | 3.520 | 3.400 | 3.470 | 3.430 |
| 76 | 453.168 | 4.640 | 6.810 | 5.660 | 7.490 |
| 77 | 475.326 | 12.420 | 11.580 | 10.390 | 9.960 |
| 78 | 475.828 | 2.990 | 2.520 | 2.690 | 3.050 |
| 79 | 476.329 | 2.960 | 2.820 | 2.840 | 2.660 |
| 80 | 477.205 | 10.090 | 11.030 | 10.830 | 10.270 |
| 81 | 478.208 | 2.320 | 2.540 | 2.450 | 2.160 |
| 82 | 479.360 | 9.890 | 10.690 | 11.620 | 11.040 |
| 83 | 480.363 | 2.970 | 3.230 | 3.500 | 3.320 |
| 84 | 499.187 | 9.680 | 10.280 | 10.180 | 9.720 |
| 85 | 500.189 | 0.000 | 2.320 | 2.300 | 0.000 |
| 86 | 518.312 | 3.480 | 5.440 | 3.710 | 4.850 |
| 87 | 521.169 | 6.880 | 7.040 | 7.120 | 6.840 |
| 88 | 543.151 | 5.960 | 6.090 | 6.130 | 5.850 |
| 89 | 588.410 | 17.230 | 16.370 | 14.030 | 12.070 |
| 90 | 589.413 | 6.010 | 5.760 | 4.930 | 4.280 |
| 91 | 601.110 | 2.730 | 2.630 | 2.710 | 2.660 |
| 92 | 614.300 | 3.970 | 3.520 | 3.860 | 4.080 |
| 93 | 615.139 | 0.000 | 0.000 | 2.740 | 3.850 |
| 94 | 617.257 | 0.000 | 2.910 | 0.000 | 2.660 |
| 95 | 689.158 | 0.000 | 0.000 | 2.630 | 3.520 |
| 96 | 701.495 | 66.250 | 52.720 | 52.640 | 50.880 |
| 97 | 701.571 | 2.310 | 2.480 | 3.060 | 2.490 |
| 98 | 702.498 | 26.540 | 21.150 | 21.250 | 20.510 |
| 99 | 703.500 | 6.120 | 4.970 | 4.970 | 4.820 |
| 100 | 773.495 | 15.510 | 20.420 | 23.170 | 21.120 |
| 101 | 812.446 | 3.350 | 4.850 | 4.470 | 3.000 |
| 102 | 813.450 | 0.000 | 3.200 | 2.650 | 53.430 |
| 103 | 814.663 | 2.620 | 2.350 | 2.600 | 2.090 |
| 104 | 815.669 | 2.510 | 0.000 | 0.000 | 6.720 |
| 105 | 816.585 | 8.270 | 6.390 | 6.670 | 6.610 |
| 106 | 851.399 | 3.480 | 7.750 | 3.930 | 3.840 |
| 107 | 927.663 | 20.850 | 17.440 | 18.610 | 11.670 |
| 108 | 929.669 | 3.620 | 3.040 | 3.240 | 3.820 |

**Supplementary Table S4.** Mean of abundance of ion levels detected by metabolomic analysis in PNT-2 treated with cfDNA Healthy, cfDNA T1/T2, cfDNA T3/T4.

|  | Ions | Control | cfDNA Healthy | cfDNA T1/T2 | cfDNA T3/T4 |
| --- | --- | --- | --- | --- | --- |
| 1 | 104.107 | 4.928 | 6.290 | 4.687 | 5.057 |
| 2 | 112.896 | 2.368 | 2.568 | 2.638 | 2.636 |
| 3 | 114.984 | 7.810 | 9.300 | 8.810 | 8.113 |
| 4 | 116.071 | 4.682 | 5.160 | 5.299 | 5.208 |
| 5 | 118.087 | 2.453 | 0.000 | 0.000 | 0.000 |
| 6 | 120.081 | 2.630 | 2.958 | 2.973 | 2.890 |
| 7 | 124.087 | 2.537 | 2.290 | 2.312 | 2.400 |
| 8 | 127.016 | 4.393 | 0.000 | 0.000 | 0.000 |
| 9 | 130.050 | 8.512 | 9.324 | 9.268 | 9.086 |
| 10 | 130.091 | 2.433 | 2.818 | 2.890 | 2.723 |
| 11 | 132.102 | 10.743 | 12.184 | 12.103 | 11.851 |
| 12 | 135.003 | 8.748 | 9.777 | 10.237 | 9.570 |
| 14 | 143.002 | 6.364 | 7.638 | 6.649 | 6.309 |
| 15 | 147.077 | 2.494 | 2.720 | 2.714 | 2.642 |
| 16 | 148.945 | 4.200 | 4.653 | 4.614 | 4.282 |
| 17 | 149.021 | 2.567 | 2.407 | 2.210 | 0.000 |
| 19 | 154.084 | 2.578 | 2.526 | 2.598 | 2.656 |
| 20 | 155.047 | 4.850 | 0.000 | 2.835 | 2.814 |
| 21 | 157.084 | 8.871 | 7.422 | 8.608 | 5.317 |
| 22 | 158.974 | 14.627 | 17.480 | 16.479 | 15.250 |
| 24 | 169.058 | 3.629 | 4.119 | 4.181 | 4.220 |
| 25 | 173.078 | 3.073 | 3.252 | 3.160 | 2.918 |
| 26 | 175.119 | 44.897 | 51.492 | 53.662 | 53.747 |
| 27 | 176.066 | 3.676 | 4.301 | 4.483 | 4.520 |
| 28 | 176.122 | 3.559 | 4.013 | 4.198 | 4.227 |
| 29 | 177.026 | 2.364 | 2.649 | 2.814 | 2.863 |
| 30 | 183.078 | 5.895 | 8.078 | 3.362 | 3.373 |
| 32 | 191.040 | 7.667 | 8.842 | 9.134 | 9.242 |
| 33 | 197.101 | 6.079 | 6.939 | 7.309 | 7.480 |
| 34 | 203.053 | 81.567 | 95.393 | 100.000 | 100.000 |
| 35 | 204.057 | 5.848 | 6.820 | 7.112 | 7.137 |
| 36 | 205.061 | 45.719 | 46.757 | 32.600 | 39.441 |
| 37 | 206.064 | 6.390 | 2.670 | 2.728 | 2.947 |
| 38 | 206.894 | 2.653 | 0.000 | 2.572 | 2.620 |
| 39 | 207.015 | 2.427 | 2.673 | 2.750 | 2.774 |
| 40 | 216.923 | 4.563 | 5.571 | 5.659 | 5.616 |
| 41 | 217.105 | 19.619 | 23.612 | 23.094 | 22.176 |
| 42 | 218.108 | 2.477 | 0.000 | 2.105 | 2.170 |
| 43 | 219.027 | 12.029 | 14.040 | 15.160 | 15.098 |
| 44 | 219.082 | 4.462 | 5.068 | 5.377 | 5.579 |
| 45 | 221.031 | 1.320 | 0.000 | 0.000 | 0.000 |
| 46 | 225.035 | 2.542 | 2.940 | 2.897 | 2.859 |
| 47 | 226.952 | 2.827 | 2.579 | 2.500 | 2.488 |
| 48 | 241.031 | 2.489 | 2.692 | 2.810 | 2.678 |
| 49 | 261.012 | 2.310 | 2.670 | 0.000 | 2.862 |
| 50 | 261.131 | 23.329 | 28.166 | 28.104 | 26.703 |
| 51 | 262.135 | 2.690 | 3.232 | 3.229 | 3.078 |
| 53 | 277.105 | 2.647 | 2.445 | 2.235 | 2.225 |
| 54 | 281.173 | 1.995 | 2.563 | 3.120 | 0.000 |
| 55 | 301.141 | 16.407 | 18.624 | 20.079 | 10.768 |
| 56 | 302.145 | 2.918 | 3.344 | 3.574 | 2.433 |
| 57 | 305.157 | 14.837 | 18.029 | 18.090 | 17.139 |
| 58 | 306.161 | 2.660 | 2.502 | 2.523 | 2.448 |
| 61 | 337.172 | 1.683 | 2.476 | 2.502 | 2.426 |
| 62 | 349.183 | 6.014 | 7.273 | 7.319 | 6.939 |
| 63 | 357.088 | 2.601 | 2.910 | 2.980 | 2.859 |
| 64 | 362.242 | 6.192 | 3.817 | 6.422 | 6.913 |
| 67 | 376.260 | 2.570 | 2.243 | 2.468 | 0.000 |
| 68 | 379.282 | 2.325 | 3.242 | 4.318 | 2.953 |
| 69 | 383.116 | 19.098 | 22.248 | 24.481 | 24.969 |
| 70 | 384.120 | 2.692 | 3.127 | 3.357 | 3.508 |
| 72 | 393.209 | 2.683 | 2.516 | 2.506 | 2.433 |
| 73 | 398.242 | 68.975 | 72.544 | 78.960 | 66.706 |
| 74 | 399.245 | 15.089 | 16.602 | 17.974 | 15.276 |
| 75 | 400.248 | 2.710 | 2.448 | 2.677 | 2.315 |
| 76 | 413.266 | 2.693 | 2.699 | 2.838 | 3.308 |
| 77 | 414.217 | 8.298 | 9.771 | 9.752 | 8.854 |
| 78 | 415.218 | 3.153 | 4.003 | 3.224 | 3.177 |
| 79 | 418.784 | 6.702 | 7.303 | 9.487 | 9.959 |
| 80 | 419.285 | 3.411 | 3.703 | 4.756 | 4.990 |
| 81 | 435.333 | 3.752 | 4.283 | 4.502 | 4.037 |
| 83 | 438.197 | 13.690 | 20.926 | 11.117 | 13.897 |
| 84 | 439.200 | 2.847 | 3.681 | 3.073 | 2.795 |
| 86 | 454.171 | 6.419 | 9.781 | 5.050 | 6.283 |
| 87 | 455.169 | 3.237 | 4.143 | 3.190 | 2.849 |
| 88 | 474.285 | 3.283 | 4.414 | 3.740 | 2.966 |
| 89 | 475.326 | 18.621 | 12.822 | 19.051 | 20.011 |
| 90 | 475.827 | 3.400 | 4.070 | 4.841 | 4.617 |
| 91 | 476.329 | 5.583 | 3.069 | 4.421 | 4.729 |
| 92 | 479.359 | 3.943 | 4.013 | 4.528 | 4.217 |
| 94 | 491.300 | 1.457 | 0.000 | 0.000 | 0.000 |
| 96 | 531.867 | 2.554 | 2.724 | 3.081 | 2.873 |
| 97 | 532.350 | 3.120 | 0.000 | 2.382 | 2.195 |
| 100 | 560.367 | 3.335 | 2.840 | 2.787 | 2.855 |
| 101 | 575.130 | 2.731 | 3.356 | 3.524 | 3.521 |
| 104 | 589.413 | 7.507 | 4.461 | 7.171 | 8.252 |
| 109 | 617.257 | 3.455 | 4.524 | 3.230 | 3.101 |
| 114 | 701.494 | 52.354 | 49.067 | 63.760 | 71.202 |
| 115 | 701.573 | 2.790 | 3.014 | 2.979 | 2.923 |
| 116 | 702.497 | 20.997 | 19.928 | 25.611 | 28.534 |
| 117 | 703.500 | 4.889 | 4.713 | 6.365 | 6.642 |
| 119 | 717.468 | 2.733 | 2.550 | 2.886 | 3.227 |
| 123 | 773.494 | 23.697 | 26.324 | 29.233 | 11.513 |
| 125 | 775.501 | 2.991 | 3.288 | 3.652 | 2.528 |
| 126 | 789.471 | 2.967 | 2.758 | 2.998 | 5.063 |
| 127 | 812.446 | 4.943 | 8.003 | 4.309 | 2.653 |
| 130 | 814.664 | 2.593 | 3.124 | 3.350 | 2.816 |
| 132 | 816.584 | 5.237 | 6.024 | 7.233 | 3.132 |
| 133 | 828.425 | 2.773 | 2.840 | 0.000 | 0.000 |
| 134 | 830.553 | 2.084 | 2.630 | 2.817 | 4.171 |
| 135 | 851.398 | 5.882 | 8.136 | 5.223 | 3.570 |
| 136 | 852.401 | 3.160 | 4.353 | 2.880 | 3.063 |
| 138 | 868.375 | 3.113 | 3.170 | 0.000 | 0.000 |
| 139 | 927.663 | 12.802 | 15.532 | 17.353 | 9.848 |
| 140 | 928.666 | 7.194 | 8.753 | 9.772 | 3.097 |
| 141 | 929.668 | 3.073 | 2.762 | 3.073 | 2.777 |
| 142 | 1040.746 | 3.110 | 2.650 | 2.647 | 0.000 |
